# Effects of dietary vitamin E on muscle vitamin E and fatty acid content in Aohan fine-wool sheep

**DOI:** 10.1186/2049-1891-4-21

**Published:** 2013-06-19

**Authors:** Kun Liu, Suyun Ge, Hailing Luo, Dubing Yue, Leyan Yan

**Affiliations:** 1 State Key Laboratory of Animal Nutrition, College of Animal Science and Technology, China Agricultural University, Beijing 100193, China

**Keywords:** Aohan fine-wool sheep, Fatty acids, Muscle, Vitamin E

## Abstract

**Background:**

Increasing the polyunsaturated fatty acid (PUFA) content and decreasing the saturated fatty acid (SFA) content of mutton can help to improve its nutritional value for consumers. Several laboratories have evaluated the effects of vitamin E on the fatty acid (FA) composition of muscle in sheep. However, little information is available on wool sheep, even though wool sheep breeds are an important source of mutton, especially in northern China where sheep are extensively farmed. The present study was designed to address the effects of vitamin E on muscle FA composition in male Aohan fine-wool sheep.

**Methods:**

Forty-two male Aohan fine-wool lambs (5 mo old) with similar initial body weight were randomly divided into seven groups and fed diets supplemented with 0 (control group), 20, 100, 200, 1,000, 2,000, or 2,400 IU/sheep/d vitamin E for 12 mo. Three lambs from each group were slaughtered to measure vitamin E and FA content in the longissimus lumborum (LL) and gluteus medius (GM) muscles.

**Results:**

Vitamin E concentrations in the LL and GM increased significantly after 12 mo of vitamin E supplementation (*P* < 0.05). However, this increase did not occur in a dose-dependent manner because the muscle vitamin E concentration was highest in the 200 IU/sheep/d group. Dietary vitamin E supplementation also caused a significant reduction in SFA content and an increase in monounsaturated FA (MUFA) content in the LL and GM (*P* < 0.05). All six doses of vitamin E significantly increased *cis*9 *trans*11-conjugated linoleic acid (*c*9*t*11-CLA) content in the LL compared with the control group (*P* < 0.05).

**Conclusions:**

Dietary supplementation with vitamin E increased muscle vitamin E content and improved the nutritional value of mutton by decreasing SFA content and increasing MUFA and *c*9*t*11-CLA contents in Aohan fine-wool sheep. These effects were greatest in sheep fed a diet containing 200 IU/sheep/d vitamin E.

## Introduction

In recent years, an increase in consumer interest in the nutritional aspects of health has resulted in the development of specific health recommendations for food components, especially of dietary fat [1]. The relationship between dietary fat and the incidence of lifestyle diseases, particularly cardiovascular disease and atherosclerosis, is well established. It is recommended that total fat and saturated fatty acids (SFAs) should not exceed 35% and 10% of the total dietary intake, respectively. Additionally, the ratio of polyunsaturated fatty acids (PUFAs) to SFAs (P/S) should be around 0.45, while that of n-6 to n-3 PUFAs should be < 4 [2]. Meat and meat products are important dietary components that provide a major source of macronutrients [3]. Therefore, increasing the PUFA content and decreasing the SFA content in meat may help to improve the nutritional value of this food type to consumers.

Some authors have demonstrated that the fatty acid (FA) profile of meat can be modified by oxidative processes and that the oxidative stability of lipid fractions was associated with their FA composition [4,5]. The susceptibility of unsaturated FAs (UFAs) to oxidation is related to the degree of unsaturation of FAs, because PUFAs are more prone to oxidation than monounsaturated FAs (MUFAs) [6]. It was also demonstrated that lipid oxidation could reduce the content of essential PUFAs (C18:2n-6 and C18:3n-3) and long-chain PUFAs (C20:5n-3 and C22:6n-3); however, oxidation of these PUFAs may be reduced by increasing the availability of natural antioxidants that could delay or inhibit oxidation [7].

Vitamin E is a major antioxidant that protects tissues from oxidative damage. It is deposited in the cell membrane where it protects the membrane PUFAs from oxidation [8]. This protective effect is carried over into the meat and can be enhanced by feeding animals with vitamin E at a level greater than that required for normal growth and reproduction. Demirel et al. reported that high dietary vitamin E supplementation provided additional protection against lipid peroxidation by increasing PUFA content and decreasing monoenoic FA content in muscle, liver, and adipose tissue [9]. Morel et al. also found that dietary vitamin E could reduce lipid oxidation in pork and in processed pork products [10]. It was also reported that vitamin E could stabilize PUFAs and was a major determinant of meat quality, particularly in ruminants [11].

Although the effects of dietary vitamin E on meat quality have been extensively studied in lambs [8,12,13], it is still necessary to determine the optimum tissue concentration for improving meat quality. The efficiency of turning dietary vitamin E into muscle vitamin E is influenced by several factors, including the dietary concentration, muscle type, and the type of vitamin E being used [14]. For these reasons, the muscle vitamin E content is highly variable leading to inconsistencies among prior studies.

Although many researchers have investigated the effects of dietary vitamin E supplementation on meat quality in meat sheep, very few studies have examined the effects of vitamin E supplementation on muscle vitamin E content in wool sheep or evaluated the changes in FA profiles, even though wool sheep are an important source of mutton. Therefore, the objectives of the present study were: 1) to examine the effects of different dietary vitamin E concentrations on muscle vitamin E content; and 2) to determine the relationship between muscle vitamin E content and FA composition in male Aohan fine-wool sheep, a common fine-wool sheep breed in China.

## Materials and methods

### Animals and management

Aohan fine-wool sheep, which were initially bred in the Inner Mongolia Autonomous Region of China, are well known for their high-grade fleece weight (16.2 kg), staple length (7.5–9.8 cm), fiber diameter (21.6–25 μm), and exceptional meat quality. We purchased 42 male Aohan lambs (5 mo old) with similar initial body weights from the Aohan fine-wool sheep breeding farm (Chifeng, Inner Mongolia Autonomous Region, China). The sheep were initially fed a diet with a forage/concentrate ratio of 6/4. The formulation developed according to the National Research Council (NRC) feeding standard is shown in Table 1[15]. The animals were housed individually, had free access to fresh, clean water, and were fed *ad libitum*.

**Table 1:** Ingredients and nutritional composition of the experimental diet

**Diet ingredients, % (DM basis)**	**Ratio**
Corn stalk pellets	60.0
Corn	24.8
Soybean meal	10.4
Wheat bran	3.2
Sodium chloride	0.4
Calcium hydrophosphate	0.8
Premix^a^	0.4
**Nutrient composition (DM basis)**	
Dry matter, %	93.56
Digestible energy, MJ/kg	8.13
Crude protein, %	10.39
Ether extract, %	8.15
Neutral detergent fiber, %	22.79
Acid detergent fiber, %	16.0
Vitamin E, IU/kg	5.08

^a^ Per kilogram of premix: 1×10^7^ IU vitamin A, 2,000 mg Cu, 9,000 mg Fe, 6,500 mg Mn, 7,500 mg Zn, 3,000 mg Mg, 250 mg I, 20 mg Se, and 30 mg Co.

Powdered vitamin E acetate (1 mg contains 1 IU vitamin E) was bought from Zhejiang Guobang Pharmaceutical Co., Ltd. (Shangyu, China) and was thoroughly mixed with the food. The sheep were divided randomly into seven groups (*n* = 6/group) and were fed diets supplemented with 0 (control), 20, 100, 200, 1,000, 2,000 or 2,400 IU/sheep/d vitamin E (E0, E20, E100, E200, E1,000, E2,000, and E2,400, respectively) for 12 mo. The dose levels were 0, 1, 5, 10, 50, 100, and 120 times higher than the NRC feeding standard [15] and were selected based on our previous findings [16]. Dry matter intake (DMI) was recorded in the last month of feeding until the sheep were slaughtered.

Three sheep of each group were randomly selected for slaughter at the end of the feeding period and samples of the longissimus lumborum (LL) and gluteus medius (GM) muscles were collected and stored at -20°C to measure vitamin E and FA content.

All of the animal procedures were conducted with approval from the China Agricultural University Animal Care and Use Committee.

### Chemical analysis

Muscle vitamin E content was measured using a commercially available vitamin E assay kit (Nanjing Jiancheng Bioengineering Institute, Nanjing, China). The principal of this kit of that, in the presence of phenanthroline, Fe^3+^ is reduced to Fe^2+^ by vitamin E. Phenanthroline then forms a complex with Fe^2+^ forming a colored adduct. Vitamin E content can be calculated using the following equation:

$$\text{Vitamin}\;E\;\text{concentration}=\frac{{\mathrm{OD}}_{U}-{\mathrm{OD}}_{B}}{{\mathrm{OD}}_{S}-{\mathrm{OD}}_{B}}\times C_{S}\times N$$

where OD_U_ = absorbance of the assay tube, OD_S_ = absorbance of the standard tube, OD_B_ = absorbance of the blank tube, C_S_ = standard concentration, and N = dilution ratio.

Muscle FA content was analyzed by gas chromatography according to the method described previously [17]. Briefly, the LL and GM were freeze-dried and 300 mg of each sample was placed into tissue culture tubes fitted with a rubber stopper and screw cap. Next, 4 mL of methanol:chloroacetyl solution (*v/v* 10:1) and 5 mL of normal hexane solution (1 mg/mL) were added to each tube. The normal hexane solution was prepared as follows: 1 g of nonadecanoic acid (Sigma, St. Louis, MO, USA) was transferred to a 1,000 mL volumetric flask and the flask was filled to a specified volume with normal hexane. The flask was covered tightly with the rubber stopper and screw cap to prevent gas leakage and was incubated in a water bath for 2 h at 80°C. After cooling to room temperature, 5 mL of 7% potassium carbonate was added, the flask was thoroughly mixed and left to stand. After the formation of distinct layers in the solution, the upper layer was used for FA analysis. FA methyl esters were analyzed by gas chromatography (HP6890) using a capillary column (HP-INNOWAX [19091N-213, 60.0 m × 320 μm × 0.5 μm], Agilent, Santa Clara, California, America). Gas chromatography was performed using the following conditions: the temperature was held at 220°C for 10 min and then increased to 250°C at a rate of 10°C/min; the temperature of the FID detector was set at 280°C; the split flow ratio was 20:1; the injection volume was 1 μL; the injection temperature was 250°C; and nitrogen (3.0 mL/min) was used as the flow gas.

### Statistical analysis

The effects of dietary vitamin E content on muscle vitamin E and FA contents were analyzed by one-way analysis of variance followed by Duncan’s new multiple range test. We used SAS 9.1 software (SAS Institute, Cary, NC, USA) for all analyses. Results are expressed as the mean ± standard error (SE). FA content was compared between the two muscles using paired *t* tests. Differences at *P* < 0.05 were considered statistically significant.

## Results

### Food intake and muscle vitamin E concentrations

The DMI and nutrient intake are shown in Table 2; there were no differences among the seven groups in these factors (*P* > 0.05). The muscle vitamin E levels are presented in Table 3. Muscle vitamin E concentrations were significantly increased by dietary vitamin E supplementation. Interestingly, the increase was not dose-dependent, because vitamin E levels were highest in the E200 group in the LL and GM. In the LL, vitamin E concentrations in the E200, E1,000, and E2,400 groups were significantly higher than that in the control group (E0). Vitamin E content was also significantly higher in the E200 group than in the E20 and E100 groups (*P* < 0.05). In the GM, the vitamin E concentration was significantly higher in the E200 group than in the other groups (*P* < 0.05) except for the E1,000 and E2,400 groups.

**Table 2:** Effects of dietary vitamin E supplementation on food intake and nutrient levels in Aohan fine-wool sheep

**Variable (kg·DM/d)**	**Treatments (IU/sheep/d vitamin E)**							***P*-value**
	**0**	**20**	**100**	**200**	**1,000**	**2,000**	**2,400**	
DMI	2.11 ± 0.11	1.86 ± 0.47	2.26 ± 0.13	2.32 ± 0.11	2.37 ± 0.09	2.15 ± 0.10	2.19 ± 0.10	0.6704
CP	0.23 ± 0.01	0.21 ± 0.05	0.25 ± 0.01	0.26 ± 0.01	0.26 ± 0.01	0.24 ± 0.01	0.24 ± 0.01	0.6704
EE	0.25 ± 0.01	0.22 ± 0.06	0.25 ± 0.02	0.26 ± 0.01	0.26 ± 0.01	0.24 ± 0.01	0.24 ± 0.01	0.6704
NDF	0.51 ± 0.03	0.45 ± 0.11	0.55 ± 0.03	0.56 ± 0.03	0.58 ± 0.02	0.52 ± 0.03	0.53 ± 0.02	0.6704
ADF	0.36 ± 0.02	0.32 ± 0.08	0.39 ± 0.02	0.40 ± 0.02	0.40 ± 0.02	0.37 ± 0.02	0.37 ± 0.02	0.6704

*DMI* dry matter intake, *CP* crude protein, *EE* ether extract, *NDF* neutral detergent fiber, *ADF* acid detergent fiber.

Data are presented as the mean ± standard error.

**Table 3:** Vitamin E content (μg/g) in the longissimus lumborum and gluteus medius at slaughter

**Item**	**Treatments (IU/sheep/d vitamin E)**							***P*-value**
	**0**	**20**	**100**	**200**	**1,000**	**2,000**	**2,400**	
LL	1.26 ± 0.27^a^	1.85 ± 0.23^ab^	2.30 ± 0.41^ab^	5.23 ± 0.94^c^	3.65 ± 1.35^bc^	3.38 ± 0.21^abc^	3.74 ± 0.12^bc^	0.0187
	(0.81-1.76)	(1.62-2.30)	(1.89-2.70)	(3.38-6.49)	(2.16-6.35)	(2.97-3.65)	(3.51-3.92)	
GM	1.77 ± 0.33^a^	2.17 ± 0.33^a^	2.71 ± 0.82^a^	7.14 ± 1.99^b^	4.19 ± 1.24^ab^	3.64 ± 0.46^a^	3.86 ± 0.08^ab^	0.0401
	(1.36-2.42)	(1.52-2.58)	(1.36-4.19)	(4.60-11.06)	(2.73-6.67)	(3.03-4.55)	(3.79-3.94)	

*LL* longissimus lumborum, *GM* gluteus medius.

Values in the same row with different superscripts differ significantly (*P* < 0.05). Data are presented as the mean ± standard error (range).

### Muscle SFA composition

Tables 4 and 5 show the muscle SFA composition in the LL and GM, respectively. In the LL, the total SFA content was significantly lower in the vitamin E-supplemented groups than in the control group (*P* < 0.05). In terms of individual SFAs, we found significant differences in C12:0, C14:0, C16:0, C18:0, and C20:0 FAs (*P* < 0.05) (Table 4). Of note, the total SFA content was lowest in the E200 group, mainly because of the lower levels of C12:0, C14:0, C16:0, C18:0, and C20:0 FAs. Similarly, the ratio of C14:0+C16:0 was also significantly lower in the vitamin E-supplemented groups, except in the E2,000 group, than in the control group (*P* < 0.05).

**Table 4:** Effects of vitamin E supplementation on saturated fatty acid content (% of total fatty acids) in the longissimus lumborum

**Item**	**Treatments (IU/sheep/d vitamin E)**							***P*-value**
	**0**	**20**	**100**	**200**	**1,000**	**2,000**	**2,400**	
C6:0	0.044 ± 0.005	0.030 ± 0.003	0.031 ± 0.011	0.033 ± 0.005	0.052 ± 0.019	0.031 ± 0.003	0.035 ± 0.007	0.5795
C8:0	0.030 ± 0.008	0.025 ± 0.004	0.023 ± 0.010	0.026 ± 0.007	0.054 ± 0.030	0.024 ± 0.001	0.025 ± 0.002	0.6088
C10:0	0.162 ± 0.010	0.131 ± 0.015	0.079 ± 0.003	0.091 ± 0.025	0.109 ± 0.042	0.136 ± 0.019	0.126 ± 0.014	0.1962
C12:0	0.125±0.010^c^	0.096 ± 0.003^abc^	0.090 ± 0.016^ab^	0.065 ± 0.014^a^	0.076 ± 0.004^ab^	0.100 ± 0.006^bc^	0.076 ± 0.007^ab^	0.0132
C14:0	2.54 ± 0.18^c^	2.09 ± 0.07^abc^	1.90 ± 0.38^abc^	1.44 ± 0.30^a^	1.69 ± 0.10^ab^	2.29 ± 0.08^bc^	1.71 ± 0.05^ab^	0.0250
C16:0	33.96 ± 2.77^c^	25.24 ± 1.59^b^	23.18 ± 0.30^ab^	19.26 ± 1.75^a^	22.77 ± 0.67^ab^	30.01 ± 1.02^c^	25.55 ± 0.40^b^	0.0001
C18:0	29.97 ± 1.14^b^	23.95 ± 2.32^a^	22.33 ± 2.69^a^	18.40 ± 0.23^a^	23.62 ± 1.02^a^	23.88 ± 2.85^a^	21.50 ± 0.52^a^	0.0213
C20:0	0.263 ± 0.012^c^	0.211 ± 0.042^bc^	0.175 ± 0.037^ab^	0.118 ± 0.012^a^	0.200 ± 0.003^bc^	0.160 ± 0.023^ab^	0.133 ± 0.003^ab^	0.0119
C22:0	0.074 ± 0.010	0.057 ± 0.011	0.047 ± 0.003	0.045 ± 0.003	0.078 ± 0.026	0.040 ± 0.002	0.046 ± 0.005	0.2117
C24:0	0.063 ± 0.010	0.056 ± 0.004	0.056 ± 0.012	0.054 ± 0.009	0.092 ± 0.040	0.049 ± 0.005	0.057 ± 0.009	0.6327
C14:0+C16:0	36.50 ± 2.92^d^	27.33 ± 1.66^bc^	25.08 ± 0.67^ab^	20.70 ± 2.05^a^	24.46 ± 0.76^ab^	32.30 ± 1.09^cd^	27.27 ± 0.42^bc^	0.0001
SFA	67.23 ± 3.33^d^	51.88 ± 1.32^bc^	47.91 ± 3.37^b^	39.54 ± 2.29^a^	48.74 ± 1.42^bc^	56.72 ± 3.74^c^	49.27 ± 0.12^bc^	0.0001

*SFA* saturated fatty acid.

Values in the same row with different superscripts differ significantly (*P* < 0.05). Data are presented as the mean ± standard error.

**Table 5:** Effects of vitamin E supplementation on saturated fatty acid content (% of total fatty acids) in the gluteus medius

**Item**	**Treatments (IU/sheep/d vitamin E)**							***P*-value**
	**0**	**20**	**100**	**200**	**1,000**	**2,000**	**2,400**	
C6:0	0.037 ± 0.007	0.037 ± 0.006	0.033 ± 0.006	0.031 ± 0.005	0.042 ± 0.012	0.037 ± 0.008	0.033 ± 0.001	0.9374
C8:0	0.040 ± 0.009	0.036 ± 0.009	0.022 ± 0.006	0.021 ± 0.002	0.051 ± 0.032	0.043 ± 0.012	0.044 ± 0.012	0.7198
C10:0	0.200 ± 0.036^b^	0.091 ± 0.010^a^	0.097 ± 0.006^a^	0.073 ± 0.008^a^	0.138 ± 0.039^ab^	0.103 ± 0.008^a^	0.187 ± 0.034^b^	0.0147
C12:0	0.152 ± 0.049	0.159 ± 0.056	0.074 ± 0.014	0.055 ± 0.013	0.071 ± 0.008	0.100 ± 0.009	0.136 ± 0.017	0.1321
C14:0	2.93 ± 0.94	2.68 ± 0.85	1.61 ± 0.29	1.16 ± 0.30	1.59 ± 0.14	2.03 ± 0.21	2.96 ± 0.38	0.1601
C16:0	32.52 ± 2.31^c^	22.89 ± 1.01^b^	23.69 ± 0.27^b^	16.56 ± 2.92^a^	21.66 ± 0.82^b^	24.36 ± 1.11^b^	23.67 ± 0.48^b^	0.0004
C18:0	25.87 ± 3.50	24.69 ± 4.49	20.08 ± 1.46	15.09 ± 3.29	19.86 ± 1.57	18.56 ± 0.29	20.38 ± 0.46	0.1444
C20:0	0.118 ± 0.010	0.171 ± 0.060	0.195 ± 0.074	0.096 ± 0.023	0.135 ± 0.010	0.108 ± 0.054	0.169 ± 0.042	0.6802
C22:0	0.043 ± 0.007	0.051 ± 0.004	0.069 ± 0.022	0.041 ± 0.008	0.066 ± 0.023	0.035 ± 0.002	0.064 ± 0.011	0.4205
C24:0	0.142 ± 0.088	0.060 ± 0.003	0.061 ± 0.010	0.054 ± 0.008	0.085 ± 0.033	0.055 ± 0.008	0.083 ± 0.017	0.6291
C14:0+C16:0	35.46 ± 1.56^c^	25.57 ± 0.65^b^	25.30 ± 0.06^b^	17.72 ± 3.21^a^	23.25 ± 0.93^b^	26.38 ± 1.32^b^	26.63 ± 0.13^b^	0.0001
SFA	62.05 ± 4.92^c^	50.87 ± 5.19^bc^	45.93 ± 1.34^b^	33.18 ± 6.47^a^	43.69 ± 2.28^ab^	45.43 ± 1.49^b^	47.73±0.58^b^	0.0057

*SFA* saturated fatty acid.

Values in the same row with different superscripts differ significantly (*P* < 0.05). Data are presented as the mean ± standard error.

In the GM, the total SFA content was significantly lower in the vitamin E-supplemented groups, except for the E20 group, than in the control group (*P* < 0.05). The total SFA content was lowest in the E200 group. Regarding individual SFAs, the C10:0 content was significantly lower in the vitamin E-supplemented groups, except in the E1,000 and E2,400 groups, than in the control group (*P* < 0.05). The C16:0 content was also significantly lower in each vitamin E-supplemented group than in the control group (*P* < 0.05) (Table 5). The relative C10:0 and C16:0 contents were lower in the E200 than in the other groups. As in the LL, the ratio of C14:0+C16:0 FAs in the GM were significantly lower in the vitamin E-supplemented groups than in the control group (*P* < 0.05), and were lowest in the E200 group.

### Muscle UFA composition

The UFA profile of the LL is shown in Table 6. The total MUFA content was significantly higher in each vitamin E-supplemented group than in the control group (*P* < 0.05), which was mainly due to the significantly higher levels of C14:1, C16:1, C18:1n9, C18:1n7, and C20:1 in the vitamin E-supplemented groups (*P* < 0.05) (Table 6). The index (C18:0+C18:1):C16:0 was significantly increased by vitamin E supplementation (*P* < 0.05), except in the E2,000 group, and was highest in E200 group. Unexpectedly, vitamin E supplementation did not affect the total PUFA content, or the n-6 and n-3 PUFA content (*P* > 0.05). For individual PUFAs, only *cis*9 *trans*11-conjugated linoleic acid (*c*9*t*11-CLA) content was significantly increased by vitamin E supplementation, except in the E20 group, compared with the control group (*P* < 0.05). *c*9*t*11-CLA content was highest in the E200 group. Vitamin E supplementation significantly increased the MUFA:SFA ratio (*P* < 0.05) but not the PUFA:SFA ratio (*P* > 0.05).

**Table 6:** Effects of vitamin E supplementation on unsaturated fatty acid content (% of total fatty acids) in the longissimus lumborum

**Item**	**Treatments (IU/sheep/d vitamin E)**							***P*-value**
	**0**	**20**	**100**	**200**	**1,000**	**2,000**	**2,400**	
C14:1	0.040 ± 0.006^a^	0.057 ± 0.007^a^	0.059 ± 0.001^a^	0.049 ± 0.010^a^	0.057 ± 0.011^a^	0.084 ± 0.011^b^	0.052 ± 0.004^a^	0.0432
C16:1	0.75 ± 0.14^a^	1.30 ± 0.08^b^	1.26 ± 0.06^b^	1.65 ± 0.10^c^	1.22 ± 0.11^b^	1.02 ± 0.18^ab^	1.21 ± 0.04^b^	0.0025
*t*11-C18:1(VA)	0.76 ± 0.14	1.32 ± 0.47	2.95 ± 1.28	1.58 ± 0.12	1.46 ± 0.55	1.12 ± 0.56	1.26 ± 0.40	0.3348
C18:1n9	23.42 ± 3.40^a^	37.79 ± 1.56^b^	38.57 ± 0.88^b^	48.17 ± 1.78^c^	35.92 ± 3.48^b^	32.56 ± 3.41^b^	39.46 ± 1.71^b^	0.0004
C18:1n7	0.91 ± 0.11^a^	1.28 ± 0.03^bc^	1.40 ± 0.05^cd^	1.42 ± 0.06^cd^	1.63 ± 0.14^d^	1.07 ± 0.08^ab^	1.28 ± 0.04^bc^	0.0005
C20:1	0.063 ± 0.004^a^	0.081 ± 0.005^ab^	0.108 ± 0.004^cd^	0.119 ± 0.013^d^	0.116 ± 0.006^d^	0.114 ± 0.004^cd^	0.094 ± 0.002^bc^	0.0002
C22:1	0.014 ± 0.002	0.016 ± 0.001	0.014 ± 0.002	0.013 ± 0.001	0.034 ± 0.014	0.016 ± 0.002	0.017 ± 0.003	0.2172
C24:1	0.033 ± 0.002	0.034 ± 0.002	0.029 ± 0.008	0.032 ± 0.005	0.052 ± 0.022	0.029 ± 0.004	0.033 ± 0.002	0.5996
MUFA	26.00 ± 3.78^a^	41.87 ± 1.53^b^	44.39 ± 1.46^b^	53.04 ± 1.76^c^	40.49 ± 2.92^b^	36.01 ± 3.59^b^	43.42 ± 1.23^b^	0.0001
C18:2n6	3.18 ± 0.23	2.75 ± 0.30	3.44 ± 1.11	3.16 ± 0.47	4.58 ± 1.81	3.07 ± 0.43	3.22 ± 0.63	0.8425
C18:3n6	0.043 ± 0.007	0.043 ± 0.010	0.064 ± 0.012	0.059 ± 0.002	0.082 ± 0.015	0.052 ± 0.004	0.055 ± 0.011	0.1371
C18:3n3	0.98 ± 0.12	0.93 ± 0.16	1.13 ± 0.26	1.06 ± 0.07	1.63 ± 0.69	1.03 ± 0.11	1.01 ± 0.20	0.7220
*c*9*t*11-CLA	0.23 ± 0.05^a^	0.36 ± 0.05^ab^	0.48 ± 0.06^bc^	0.66 ± 0.07^d^	0.49 ± 0.05^bc^	0.63 ± 0.05^cd^	0.43 ± 0.01^b^	0.0006
*t*10*c*12-CLA	0.038 ± 0.004	0.044 ± 0.003	0.041 ± 0.008	0.051 ± 0.011	0.060 ± 0.013	0.047 ± 0.004	0.044 ± 0.003	0.5261
C20:2	0.41 ± 0.04	0.36 ± 0.03	0.44 ± 0.08	0.41 ± 0.03	0.63 ± 0.20	0.41 ± 0.04	0.45 ± 0.03	0.4296
C20:4n6	1.47 ± 0.14	1.39 ± 0.14	1.64 ± 0.47	1.59 ± 0.24	2.63 ± 1.17	1.62 ± 0.32	1.65 ± 0.32	0.6994
C20:5n3	0.057 ± 0.004	0.056 ± 0.002	0.064 ± 0.014	0.056 ± 0.004	0.098 ± 0.034	0.061 ± 0.011	0.065 ± 0.015	0.5303
C22:4n6	0.16 ± 0.01	0.15 ± 0.01	0.17 ± 0.04	0.17 ± 0.03	0.23 ± 0.09	0.15 ± 0.02	0.18 ± 0.03	0.8418
C22:5n3	0.17 ± 0.01	0.16 ± 0.01	0.19 ± 0.04	0.18 ± 0.02	0.29 ± 0.11	0.18 ± 0.03	0.17 ± 0.03	0.5632
C22:6n3	0.023 ± 0.002	0.021 ± 0.003	0.032 ± 0.011	0.034 ± 0.003	0.050 ± 0.018	0.032 ± 0.007	0.040 ± 0.012	0.4236
PUFA	6.77 ± 0.47	6.25 ± 0.52	7.70 ± 1.98	7.42 ± 0.87	10.77 ± 4.10	7.27±1.02	7.32 ± 1.24	0.7211
n-6 PUFA	4.85 ± 0.37	4.33 ± 0.40	5.32 ± 1.63	4.98 ± 0.74	7.52 ± 3.08	4.89±0.78	5.10 ± 0.98	0.7900
n-3 PUFA	1.23 ± 0.11	1.16 ± 0.16	1.42 ± 0.32	1.32 ± 0.09	2.06 ± 0.86	1.30±0.16	1.29 ± 0.25	0.6818
(18:0+18:1)/16:0	1.66 ± 0.25^a^	2.58 ± 0.23^bc^	2.81 ± 0.03^c^	3.68 ± 0.38^d^	2.75 ± 0.08^c^	1.96±0.12^ab^	2.49 ± 0.10^bc^	0.0002
*c*9*t*11-CLA/VA	0.30 ± 0.01	0.34 ± 0.11	0.24 ± 0.09	0.43 ± 0.08	0.67 ± 0.45	0.84±0.30	0.50 ± 0.24	0.5607
n-6/n-3	3.96 ± 0.17	3.80 ± 0.34	3.61 ± 0.34	3.74 ± 0.36	3.73 ± 0.15	3.73±0.14	3.95 ± 0.24	0.9597
MUFA/SFA	0.39 ± 0.08^a^	0.81 ± 0.05^bc^	0.94 ± 0.10^c^	1.35 ± 0.12^d^	0.83 ± 0.04^bc^	0.65±0.10^b^	0.88 ± 0.03^bc^	0.0001
PUFA/SFA	0.100 ± 0.002	0.120 ± 0.010	0.168 ± 0.054	0.191 ± 0.030	0.226 ± 0.092	0.130±0.022	0.149 ± 0.025	0.4786

*VA* trans vaccenic acid, *MUFA* monounsaturated fatty acid, *CLA* conjugated linoleic acid, *PUFA* polyunsaturated fatty acid, *SFA* saturated fatty acid.

Values in the same row with different superscripts differ significantly (*P* < 0.05). Data are presented as the mean ± standard error.

The UFA profile of GM is presented in Table 7. Vitamin E supplementation significantly increased the total MUFA content, mainly because of changes in C18:1n9 (*P* < 0.05). The (C18:0+C18:1):C16:0 ratio was significantly higher in the E200 group than in the control group (*P* < 0.05). However, there were no significant differences in total PUFA, individual PUFA, or in the MUFA:SFA and PUFA:SFA ratios in the GM (*P* > 0.05).

**Table 7:** Effects of vitamin E supplementation on unsaturated fatty acid content (% of total fatty acids) in the gluteus medius

**Item**	**Treatments (IU/sheep/d vitamin E)**							***P*-value**
	**0**	**20**	**100**	**200**	**1,000**	**2,000**	**2,400**	
C14:1	0.048 ± 0.009	0.067 ± 0.010	0.100 ± 0.026	0.099 ± 0.025	0.074 ± 0.009	0.085 ± 0.007	0.099 ± 0.006	0.2013
C16:1	0.90 ± 0.16	1.30 ± 0.14	1.54 ± 0.03	1.78 ± 0.08	1.44 ± 0.14	1.59 ± 0.12	1.39 ± 0.41	0.1149
*t*11-C18:1(VA)	0.58 ± 0.06	1.26 ± 0.21	1.60 ± 0.30	2.05 ± 1.10	1.58 ± 0.12	1.36 ± 0.48	1.22 ± 0.51	0.6066
C18:1n9	28.50 ± 4.16^a^	43.08 ± 1.68^bc^	41.51 ± 0.68^bc^	52.06 ± 7.72^c^	38.60 ± 3.87^ab^	42.74 ± 0.92^bc^	39.70 ± 0.65^ab^	0.0238
C18:1n7	1.03 ± 0.15	1.56 ± 0.17	1.45 ± 0.07	1.74 ± 0.04	1.83 ± 0.24	0.99 ± 0.45	1.47 ± 0.05	0.0951
C20:1	0.080 ± 0.012	0.154 ± 0.059	0.091 ± 0.021	0.139 ± 0.023	0.118 ± 0.003	0.101 ± 0.003	0.151 ± 0.028	0.3833
C22:1	0.014 ± 0.001	0.020 ± 0.003	0.026 ± 0.006	0.017 ± 0.004	0.030 ± 0.011	0.023 ± 0.006	0.027 ± 0.004	0.4738
C24:1	0.030 ± 0.004	0.029 ± 0.003	0.046 ± 0.015	0.124 ± 0.096	0.046 ± 0.015	0.039 ± 0.016	0.061 ± 0.023	0.6387
MUFA	31.19 ± 4.49^a^	47.46 ± 1.64^bc^	46.36 ± 0.46^b^	58.01 ± 7.12^c^	43.71 ± 3.78^b^	46.93 ± 0.33^bc^	44.12 ± 0.70^b^	0.0067
C18:2n6	2.94 ± 0.40	3.11 ± 0.40	3.31 ± 0.80	3.87 ± 1.16	4.48 ± 1.62	3.19 ± 0.55	3.39 ± 0.10	0.8817
C18:3n6	0.044 ± 0.007	0.057 ± 0.008	0.065 ± 0.008	0.085 ± 0.017	0.084 ± 0.024	0.065 ± 0.011	0.088 ± 0.010	0.2496
C18:3n3	0.79 ± 0.12	0.86 ± 0.16	0.89 ± 0.18	0.95 ± 0.15	1.27 ± 0.35	0.96 ± 0.20	0.94 ± 0.06	0.7183
*c*9*t*11-CLA	0.36 ± 0.08	0.47 ± 0.04	0.65 ± 0.04	0.84 ± 0.22	0.91 ± 0.18	0.57 ± 0.03	0.55 ± 0.18	0.1057
*t*10*c*12-CLA	0.034 ± 0.004	0.042 ± 0.004	0.060 ± 0.015	0.060 ± 0.015	0.059 ± 0.011	0.064 ± 0.012	0.063 ± 0.007	0.3541
C20:2	0.41 ± 0.03	0.44 ± 0.07	0.47 ± 0.07	0.51 ± 0.03	0.74 ± 0.29	0.49 ± 0.08	0.51 ± 0.02	0.6071
C20:4n6	1.73 ± 0.22	1.75 ± 0.19	1.73 ± 0.17	1.94 ± 0.26	2.92 ± 1.29	1.77 ± 0.29	2.03 ± 0.18	0.6885
C20:5n3	0.061 ± 0.007	0.064 ± 0.009	0.069 ± 0.011	0.072 ± 0.007	0.104 ± 0.027	0.077 ± 0.015	0.079 ± 0.006	0.3883
C22:4n6	0.17 ± 0.01	0.18 ± 0.03	0.19 ± 0.04	0.21 ± 0.04	0.34 ± 0.15	0.19 ± 0.03	0.23 ± 0.01	0.5694
C22:5n3	0.19 ± 0.02	0.20 ± 0.03	0.22 ± 0.04	0.23 ± 0.03	0.32 ± 0.12	0.22 ± 0.02	0.23 ± 0.01	0.6141
C22:6n3	0.031 ± 0.002	0.030 ± 0.004	0.049 ± 0.009	0.053 ± 0.015	0.066 ± 0.030	0.049 ± 0.014	0.043 ± 0.006	0.6058
PUFA	6.76 ± 0.77	7.20 ± 0.90	7.71 ± 1.29	8.81 ± 1.92	11.29 ± 4.09	7.64 ± 1.22	8.15 ± 0.14	0.7027
n-6 PUFA	4.88 ± 0.59	5.10 ± 0.60	5.30 ± 1.00	6.10 ± 1.48	7.82 ± 3.09	5.22 ± 0.88	5.74 ± 0.20	0.8001
n-3 PUFA	1.07 ± 0.13	1.15 ± 0.20	1.23 ± 0.24	1.31 ± 0.21	1.76 ± 0.53	1.31 ± 0.25	1.29 ± 0.06	0.6631
(18:0+18:1)/16:0	1.74 ± 0.14^a^	3.09 ± 0.21^a^	2.73 ± 0.03^a^	4.67 ± 1.13^b^	2.85 ± 0.11^a^	2.63 ± 0.13^a^	2.65 ± 0.04^a^	0.0155
*c*9*t*11-CLA/VA	0.62 ± 0.12	0.41 ± 0.10	0.44 ± 0.10	0.70 ± 0.40	0.57 ± 0.10	0.64 ± 0.32	0.71 ± 0.31	0.9515
n-6/n-3	4.58 ± 0.27	4.52 ± 0.29	4.34 ± 0.27	4.54 ± 0.36	4.21 ± 0.39	4.06 ± 0.27	4.47 ± 0.35	0.8807
MUFA/SFA	0.52 ± 0.11	0.96 ± 0.12	1.01 ± 0.03	2.01 ± 0.70	1.00 ± 0.07	1.04 ± 0.04	0.93 ± 0.03	0.0556
PUFA/SFA	0.111 ± 0.018	0.144 ± 0.023	0.170 ± 0.034	0.280 ± 0.059	0.268 ± 0.110	0.170 ± 0.033	0.171 ± 0.002	0.2547

*VA* trans vaccenic acid, MUFA *monounsaturated fatty acid*, *CLA* conjugated linoleic acid, *PUFA* polyunsaturated fatty acid, *SFA* saturated fatty acid.

Values in the same row with different superscripts differ significantly (*P* < 0.05). Data are presented as the mean ± standard error.

Finally, we compared the effects of dietary vitamin E supplementation on FA deposition on FA concentration between the GM and LL. However, we found no significant differences in any of the FA parameters between the two muscles (*P* > 0.05).

## Discussion

### Muscle vitamin E concentrations

Previous studies have demonstrated increases in tissue α-tocopherol concentrations following dietary vitamin E supplementation in lambs [18,19]. Liu et al. fed steers with diets supplemented with 250, 500 or 2,000 mg all-rac-α-tocopherol acetate/calf/d and reported that the mean muscle α-tocopherol concentrations were 1.39, 2.27 and 4.95 μg/g, respectively, in the LL, semimembranosus, and GM [20]. Lauzurica et al. reported that α-tocopherol concentrations in meat increased with increasing dietary vitamin E content [21]. Kasapidou et al. also reported that the muscle α-tocopherol content increased with increasing dietary vitamin E content, and that muscle vitamin E concentrations were positively correlated with dietary vitamin E levels [8,13]. In our study, muscle vitamin E content increased significantly with increasing dietary vitamin E content, although the relationship was not dose-dependent because the muscle vitamin E content in the LL and GM was greatest in the E200. Muscle vitamin E content was lower in the E1,000, E2,000, and E2,400 groups than in E200, probably because the uptake and deposition of vitamin E were saturated at E200. It has been reported that, when animal diets are supplemented with ‘supranutritional’ vitamin E levels, the effects of dietary vitamin E on its tissue content diminishes [13]. Slow hydrolysis was also reported as a cause of low plasma tocopherol levels in animals fed supranutritional levels of α-tocopherol acetate [22].

The duration of dietary supplementation is another factor that influences α-tocopherol deposition. In general, greater dietary vitamin E content and/or longer supplementation time are associated with higher α-tocopherol concentrations in meat [23]. The muscle vitamin E content in our study was higher than that in other studies, possibly because our study was conducted over 12 mo or because we used a different animal model. Álvarez et al. reported lower semimembranosus vitamin E concentrations in younger lambs fed a diet supplemented with vitamin E for only 37 d [12]. However, in the present study, muscle vitamin E levels were highly variable among sheep in the same group (Table 3). Wide variability was also reported by other researchers. Lynch et al. reported that α-tocopherol concentrations in beef skeletal muscle ranged from 2.50 to 6.19 μg/g in cattle given 300 mg/cow/d all-rac-α-tocopheryl acetate compared with 0.70–2.92 μg/g in the control group [24]. O'Grady et al. also reported that muscle vitamin E levels ranged between 1.35 and 2.73 μg/g in the longissimus in cattle fed with 300 mg all-rac-α-tocopheryl acetate/kg feed [25]. Meanwhile, Álvarez et al. reported that muscle vitamin E content differed by almost two-fold in lambs receiving the same dietary vitamin E content [12]. Although there is no clear explanation for this variation, Kasapidou et al. suggested that differences in the ability of individual lambs to metabolize vitamin E might play a key role in this phenomenon [8]. The small number of animals used in these studies might also contribute to the high variability in muscle vitamin E content [26]. Nevertheless, greater understanding of the mechanisms controlling the uptake and metabolism of vitamin E is necessary to explain the differences observed among prior studies.

### Muscle FA profile

SFAs are implicated in the etiology of several diseases, and there is a strong positive correlation between SFA intake and the rate of coronary artery heart disease [27]. Grundy and Denke also reported that high dietary levels of long-chain SFAs increase plasma cholesterol level whereas high levels of MUFAs and PUFAs do not [28]. However, not all SFAs have equivalent effects. It was suggested that C14:0 and C16:0 exerted differential, dose-dependent effects on cholesterol and lipoprotein metabolism, even in animals fed low-cholesterol diets, while C18:0 did not appear to have such an effect [29]. It was also reported that high concentrations of palmitic acid (C16:0) were toxic to mitochondria [30] and that the concentration of stearic acid (C18:0) was positively correlated with the odor and flavor intensity of mutton [31]. In this study, the C16:0, C14:0+C16:0, and total SFA levels in the LL and GM, and C12:0, C14:0, and C18:0 levels in the LL were significantly lower in the vitamin E-supplemented groups than in the control group (*P* < 0.05), which is consistent with the results of other studies [7,32]. In our previous study, 24 Boer male goats were fed diets supplemented with 0, 80, 320 and 880 IU/goat/d vitamin E for 5 mo, which tended to decrease the C18:0 and total SFA levels, especially in the 320 IU/goat/d group [33]. However, our results did not agree with those of another study in which vitamin E supplementation (45 mg/lamb/d) significantly increased intermuscular fat stearic acid (C18:0) content but did not affect other FAs [34]. Salvatori et al. also studied the effects of vitamin E supplementation on FA composition of lamb meat and found no differences between the dietary supplementation groups [35]. The reasons for these discrepancies remain unclear, and warrant further studies.

In our study, muscle total MUFA and C18:1n9 contents were significantly higher in the vitamin E-supplemented groups than in the control group (*P* < 0.05). The higher tissue MUFA content might be partially related to the activation of their biosynthesis in the Δ^9^d-catalysed reaction. Δ^9^d also catalyzes the tissue synthesis of *c*9*t*11-CLA from *trans*11-C18:1 (*t*11-C18:1) [36]. In this study, Δ^9^d activity was estimated by calculating the product/substrate ratios of Δ^9^d-catalysed reactions (i.e. MUFA/SFA and *c*9*t*11-CLA/*t*11-C18:1) [37]. The MUFA/SFA ratio in the LL was higher in the vitamin E-supplemented groups, indicating changes in Δ^9^d activity; however, we found no differences in the muscle *c*9*t*11-CLA/*t*11-C18:1 among the experimental groups. The vitamin E-supplemented diet significantly affected the MUFA/SFA index but not the *c*9*t*11-CLA/*t*11-C18:1 index, which is somewhat surprising because both indices should reflect Δ^9^d activity. A possible explanation for this inconsistency is that CLA formation and MUFA biosynthesis might be catalyzed by different Δ^9^d isoforms that are regulated by different mechanisms [36]. Other animal species were reported to express more than one Δ^9^d isoform [38], but the number of Δ^9^d isoforms in ruminant tissues has not been clarified. To date, only one isoform has been identified in cattle and sheep [39]. However, in a recent study using bovine tissues, two Δ^9^d-immunoreactive bands at close molecular weights were found, which might indicate the presence of more than one Δ^9^ desaturase proteins [36]. Total MUFA content in the GM was also higher in the vitamin E-supplemented groups because of the higher MUFA/SFA ratio, although the differences among the experimental groups were not significant (*P* = 0.0556). As in the LL, dietary vitamin E supplementation did not affect the *c*9*t*11-CLA/t11-C18:1 index in the GM. Bonanome and Grundy reported that only C16:0 increases blood cholesterol levels, whereas C18:0 does not, and C18:1 decreases blood cholesterol levels [40]. Because these molecules represent the majority of FAs, the ratio of (C18:0+C18:1):C16:0 could help to evaluate the possible clinical effects of different lipids. As shown in Tables 6 and 7, the (C18:0+C18:1):C16:0 ratio in the LL was significantly increased by vitamin E supplementation (*P* < 0.05), and was significantly higher in the E200 group than in the control group in the GM (*P* < 0.05).

We found no significant differences in muscle PUFA content between the vitamin E-supplemented groups and the control group (*P* > 0.05). These findings are consistent with those reported by Kasapidou et al. who found that vitamin E levels did not affect phospholipid content in the muscle or FA composition, and that low vitamin E levels were sufficient to protect lipids from oxidation in sheep [8,13]. However, in other studies, PUFA content in meat was affected by vitamin E. For example, it was reported that lambs with muscle vitamin E concentrations of 0.27 mg/g had lower PUFA content in muscle than lambs with muscle vitamin E concentrations of 0.52 mg/g [9]. Álvarez et al. also reported that increasing the concentration of α-tocopherol in meat via dietary supplementation protected against PUFA oxidation [7]. The differences in findings reported to date suggest to us that further studies are needed to better understand how vitamin E affects the PUFA composition in meat. Regarding individual PUFAs, only *c*9*t*11-CLA content in the LL was significantly higher in the vitamin E-supplemented groups than in the control group (*P* < 0.05). This is consistent with the findings reported by Gabryszuk et al. who found that dietary supplementation with Se, Zn, and vitamin E improved the lipid profile and increased the concentration of CLA in the meat of Polish Merino ram-lambs [41]. Chen et al. also reported that diets supplemented with soybean oil and vitamin E increased the *c*9*t*11-CLA content in lamb meat [42].

It was notable that the dietary vitamin E content of 200 IU/sheep/d seemed to be the best dose in this study because it achieved the highest muscle vitamin E content, the lowest SFA content, and the highest MUFA and *c*9*t*11-CLA contents. In our previous study, we found that semen quality, as well as the antioxidant capacity of the testicular cell membrane and mitochondria of Aohan fine-wool sheep showed the greatest improvements when the diet was supplemented with 200 IU/sheep/d vitamin E [16]. These results suggest that this level of vitamin E supplementation has particularly beneficial health effects and provides the basis for future studies aimed at investigating the benefits of dietary supplementation with this antioxidant in sheep.

## Conclusions

Overall, the present study showed that dietary vitamin E supplementation increases muscle vitamin E content in sheep. Dietary vitamin E also reduced SFA content and increased MUFA and *c*9*t*11-CLA contents in the muscle of Aohan fine-wool sheep. These effects of vitamin E were greatest in sheep fed a diet supplemented with 200 IU/sheep/d vitamin E.

## Competing interests

The authors declare that they have no competing interests in relation to this study.

## Authors’ contributions

KL carried out the statistical analysis and drafted the manuscript. SYG participated in the chemical analysis. HLL conceived the study, participated in its design and coordination, and helped draft the manuscript. DBY and LYY performed the animal experiments. All authors read and approved the final manuscript.
